# Seismic Behavior of Extended End-Plate Connections Subjected to Cyclic Loading on the Top-Side of the Column

**DOI:** 10.3390/ma13173724

**Published:** 2020-08-23

**Authors:** Liang Luo, Jiangui Qin, Dongzhuo Zhao, Zhiwei Wu

**Affiliations:** School of Civil Engineering and Transportation, South China University of Technology, Guangzhou 510641, China; luoliang2018go@163.com (L.L.); zdcom2@126.com (D.Z.); wzw13926155919@163.com (Z.W.)

**Keywords:** cyclic loading, hysteretic performance, finite element modeling, semi-rigid connection, extended end-plate

## Abstract

The extended end-plate connections provide excellent performance in resisting seismic loads in high-risk areas. Most scholars’ experiments and finite element studies on this type of joint are focused on the method of applying displacement loads on the beam tip, while the method of applying displacement on the column side has not been the subject of further study. However, the load transmission mechanism of this type of connection is not completely consistent in actual engineering, as the design concept of “strong column weak beam” does not apply to all joints. Therefore, in this paper, the lateral displacement of the applied column is used to simulate the seismic horizontal force to study the mechanical properties of the connection joints of the “weak column and strong beam” under the limit state of earthquake action. Based on the two internal columns (IC-EP1/2) and two edge columns (EC-EP1/2), the failure modes, strength, stiffness, moment–rotation curve, skeleton curve, ductility, and energy dissipation of this type of connection were studied. Experiment results indicated that this type of connection features semi-rigid and partial strength joints. The connection rotation angle of all specimens in the test exceeds 0.05 rad, which suggests it is an ideal seismic joints. Besides, the relationship between the thickness of the end-plate and the diameter of the bolt has a greater impact on the failure mode of the joint. The finite element (FE) analysis models were established for the above connection. The numerical model was validated against experimental results and showed acceptable consistency.

## 1. Introduction

The steel structure has been widely used in the building due to its favorable stiffness and strength, especially in the seismic design of high-rise buildings. Steel frames are easy and time-efficient to assemble, and good performance can be expected at a relatively low cost [1]. As an important part of the structure, the beam–column joints were spliced together by welding in the early days. These joints were designed to be rigidly connected, which has flaws including complex configuration, a long construction period, and being difficult to maintain and repair at later stages. Most importantly, the welding residual stress cannot be ignored, as it makes brittle failure more likely to occur. In the 1990s, in Northridge and Kobe, a large number of buildings were constructed with steel frames, in which the beam–column joints were welded connections. Post-earthquake investigations [2,3,4] found that welded joints all had weld fractures. When the layer angular displacement has not yet reached the design ductility requirements, the through-weld fracture has already occurred at the joint, which makes the entire joint brittle and further leads to the failure of the whole structure.

The two earthquakes mentioned above have attracted the attention of researchers and technicians. The codes for newly designed steel structure buildings no longer recommend the use of fully welded joints as the main force structure, which is solved by high-strength bolt connections. The bolt connection joints can exhibit better deformation capacity and ductility. When various high-strength bolt connection forms with semi-rigid joints are designed and adopted, discontinuity research problems also occur between beams and columns. The extended end-plate bolt connection is a typical semi-rigid connection in actual engineering. Chen et al. [1] believe that when the frame structure adopts this kind of connection, it can increase the damping of the structure, extend the period, reduce the amplitude, and thus mitigate the earthquake damage.

The traditional steel frame design of the beam–column connection simplifies an ideal hinge or complete rigid connection [5], which no longer conforms to actual engineering. Most of the research on the moment–rotation characteristics of actual semi-rigid connections is to load the beam tip to simulate the action of the seismic force. Experiments and research rarely use column top-side loading schemes. Krishnamurthy et al. [6] first proposed the use of a flexible bolt connection; A N Sherbourne et al. [7] and Abidelah et al. [8] conducted three-dimensional finite element analysis of the end-plate connection joint to verify the feasibility of modeling. The flexible bolted connection provided the required strength, ductility, and rigidity to increase the safety for steel structure buildings. Bing Guo et al. [9] analyzed the stiffness and strength characteristics of bolted end-plate connections. It was concluded that the cyclic loading results for these connections need to be reviewed in seismic zones. Popov et al. [10] conducted beam tip loading tests on 18 beam–column connections to study the cyclic behavior of the connection to get an acceptable relationship between these methods. Grimsmo et al. [11] considered the stiffness of the panel zone of the intermediate column joints in the end-plate connection. The moment–rotation relationship is considered a very complex relationship between the connected members of the connection, which can deeply describe the behavior of connections. Meanwhile, Ioannides et al. [12] and Aribert et al. [13] used rotating springs to realize the semi-rigid characteristic connection research, but their studies focused on determining the case of welded connections failure to find the alternative bolted connections used in seismic zones with traditional parameters. Gang Shi et al.’s [14] models were subjected to monotonic and cyclic loads in order to determine the influence of these parameters, understand the connection response, and generate useful data that can be used in the nonlinear analysis of frames with flexible connections in seismic zones. Brando et al. [15] proposed a simple and effective formula for aluminum structures so that the joint strength can be predicted more reliably. Mostafa Radmehr [16] studied the moment–rotation behavior of the joints by considering the seismic uncertainty. Most previous studies applied displacement loads on the beam tip, while few studies applied horizontal loads on the column position. Changes in the loading position affect the force transmission mechanism and mechanical behaviors of the joints. The currently studied column position loading scheme is more in line with the real mechanical performance of some engineering structures under the condition of “weak column and strong beam”.

As mentioned above, for most of the research on extended end-plates, beam tip loading is used to simulate the action of seismic force, and there are few tests and studies on the column top-side loading. In this experiment, the column is designed to generate displacement and participate in energy consumption, so that the beam–column joint is not in the state of “strong column and weak beam”. These codes [17,18,19] require the *M–θ* relationship of the joints as the design basis. Through a series of indicators such as the initial rotation stiffness, bearing capacity, ductility, energy dissipation capacity, and failure mode of the joint, we can comprehensively evaluate the connection performance. The purpose of this paper is to supplement the mechanical properties of some actual engineering structures, which no longer meet the “strong column and weak beam” criterion under earthquake action. In this research, representative tests have been carried out to study the seismic performance of connections. Meanwhile, because testing steel structure connections is expensive, time-consuming, and the instrument test itself has a significant test error factor [20], 3D nonlinear finite element models were established to verify the experimental results.

## 2. Test Program

### 2.1. Test Overview

Under the action of an earthquake, since the test control index axial compression ratio is 0.3, the axial force of the joint column remains constant, and the bending moment in the joint changes alternately, so it can be simplified as the constant axial force of the column, and the joint cyclic bending moment is used for research. Most of the previous test designs were based on the “strong column, and weak beam” design concept, in which the design of the column connecting the joints is in the state of rigid body and elasticity. In reference to [21,22], the relative rotation of the column also verifies that the displacement of the column is negligible during the measurement process. In actual engineering, the cast-in-situ floor and frame beam form a T-shaped beam under the positive moment, which increases the area of the compression zone of the frame beam; and the outer frame column has a short column effect due to the problem of window opening between the filled walls between the columns, resulting in shear failure. The above series of reasons are the behaviors of strengthening beams or weakening columns, which makes it difficult to realize the design concept of “strong columns and weak beams” under the earthquake mechanism of the frame structure. The research in this paper is based on the above reasons, allowing the column part to incur large deformation to produce joint moments, in order to study the mechanical behaviors of the typical beam–column joints in the frame under the inelastic state. The experimental scheme uses cyclic displacement on the top-side of the column to simulate the earthquake action.

### 2.2. Design of Test Specimens

#### 2.2.1. Size Design Criteria

According to the existing steel structure design specifications, the reasonable design of each part in the end-plate connection joint can meet the research purpose of the experiment.

**Figure 1:**
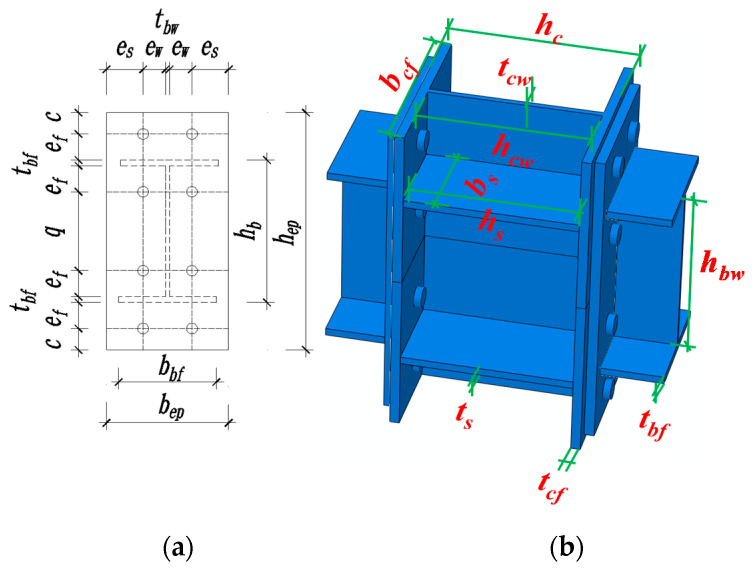
Design information: (**a**) End-plate connection parameters; (**b**) Joint parameter information.

**Figure 2:**
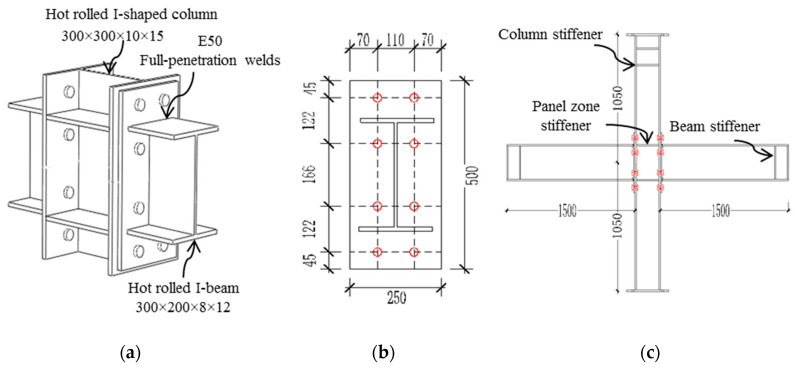
Details of joint specimens. (mm): (**a**) Detail drawing of the connection; (**b**) Layout of the end-plate and bolts; (**c**) Overall size of the intermediate column joints.

1. End-plate
  (1) End-plate height: *h_ep_* = *h_b_* + 2 (*e_f_* + *c*). As shown in Figure 1a, where *h_ep_* and *h_b_* are the height of the end-plate and the height of the beam, respectively. *e_f_* is the distance from the top row of bolts to the outer edge of the beam flange surface, and *c* is the distance from the top row of bolts to the outer edge of the end-plate. *e_f_* and *c* are 55 mm and 45 mm from the end-plate of the test specimen.
  (2) End-plate width: *b_ep_* = *t_bw_*+2 (*e_s_* + *e_w_*) and *b_bf_* ≤ *b_ep_* ≤ *b_cf_*. As displayed in Figure 1a, where *b_ep_*, *b_bf_*, and *b_cf_* are the end-plate width, beam flange width, and column flange width, respectively. In general, when the beam flange width can meet the margin requirements of bolt arrangement, the setting of *b_ep_* ≥ *b_bf_* is very appropriate. Each parameter satisfies the above design formula. For specific values, refer to Figure 2b.
  (3) End-plate thickness: In accordance with the “American Steel Structure Design Manual”, the end-plate thickness should not be less than 12 mm. The end-plate thickness should range from 12 to 30 mm. The thickness of the end-plate in this experiment is 16 mm.
2. Bolt
  (1) Bolt arrangement: Refer to the relevant methods in the American Steel Structure Design Manual. The bolt arrangement requires that the bolts of the end-plate connection should be symmetrically arranged. Two columns of bolts can be used in preference—that is, there are only two bolts in each row. Set a row of bolts on the inside and outside of the beam flange, respectively; according to actual needs, several rows of bolts can be added on the inner side of the flange.
  (2) Bolt selection: We give priority to adopt a high-strength bolt friction-type connection, the diameter is usually 16–30 mm. In this test, two commonly used bolts, M20 and M24, are selected.
3. Panel zone
  (1) Column web: The “Chinese Code for Steel Structures” and “Chinese Code for Seismic Resistance” put forward the requirements for the thickness of the column webs based on the stability of the panel zone: *t_cw_* ≥ (*h_bw_* + *h_cw_*)/90. As exhibited in Figure 1b, where *t_cw_*, *h_bw_*, and *h_cw_* are the thickness of the column web, the height of the beam web, and the height of the column web, respectively.
  (2) Column flange: In this experiment, due to the difference in shear value at the specimen panel zone, the end-plate is used as the intermediate column connection, and the column flanges on both sides of the panel zone are locally thickened. The edge column joints cannot consider this issue.
  (3) Column web stiffener: The column web stiffener meets the conditions of *t_s_* ≥ *t_bf_*, *h_s_* = *h_c_* − 2*t_cf_* and *b_s_* = 0.5 (*b_c__f_* − *t_cw_*); that is, the thickness of the stiffener is not less than the thickness of the beam flange, and the width of the stiffener reaches at least the edge of the beam flange. The column web stiffeners are aligned with the beam flange in the thickness direction. As presented in Figure 1b, where *t_s_*, *t_bf_*, *t_cf_*, and *t_cw_* are the column web stiffener thickness, beam flange thickness, and column flange thickness, *h_s_* and *b_s_* are column web stiffener height and width, and *h_c_* and *b_c_* are column section height and width.

#### 2.2.2. Specimen Configuration

Four full-scale steel beam-column extended end-plate bolt connection joint specimens were designed during the test. Variation parameters include joint type, end-plate thickness, and bolt diameter. The specimens are summarized in Table 1. Specimen numbers are IC-EP1, IC-EP2, EC-EP1, and EC-EP2; IC and EC represent the intermediate column and edge column joints, respectively; EP stands for the end-plate connection without stiffeners. The beams and columns are connected by 10.9-grade M20 friction type high-strength bolts. At the factory, the connected member was prepared by sandblasting to obtain the friction surface of the friction coefficient of 0.44. The high-strength bolt is tightened by the torque method. The initial tightening torque and final tightening torque of 10.9 grade M20 and M24 high-strength bolts are 280 N·m and 446 N·m, 400 N·m, and 760 N·m, respectively, while the gap between the bolt hole and the bolt is 2 mm.

The basic configuration of the joint is shown in Figure 2. The flanges on both sides of the intermediate column are connected to the beam, as shown in Figure 2c, while the flanges on one side of the edge column are connected to the beam. The beams and columns of the four test specimens all adopt hot-rolled I-shaped sections. The beam and column dimensions are 300 × 200 × 8 × 12 mm and 300 × 300 × 10 × 15 mm, respectively. According to the position of the reverse bending point of the frame model, the column height is 2100 mm, and the beam length is 1500 mm. The steel strength grades of beam–column joints and all components are Q345B. The size information and bolt hole layout of the extended end-plate are shown in Figure 2b. The end-plates and beams are connected by fully penetrating butt welds. All welds in the test specimen are first-grade welds, and the welding rod uses E50. All welding work is completed in the factory, and the design, processing, and production of the test specimens are in accordance with the requirements of relevant codes. Considering the concentration effect of stress, stiffeners are set on the top-side of the column and the beam tip, the distance between the stiffeners is 150 mm, the stiffeners at the panel zone are centered on the flange of the beam, and the three corners are welded. The thickness of all the stiffeners is 12 mm. The column axial compression ratio is 0.3, and the column axial force *F_c_* remains constant throughout the test, *F_c_* = 0.3*f_c_A_c_*, where *f_c_* takes the nominal yield strength of Q345B steel 345 Mpa, and *A_c_* is the column section area. Before the test displacement load, according to the design standard [23], the axial force of 1250 kN should be applied to the top of the column.

**Figure 3:**
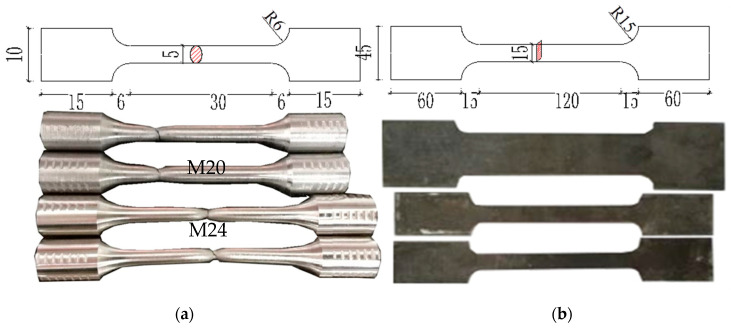
Design dimensions of material test samples (mm): (**a**) Bolt material test samples; (**b**) Steel sheet test specimens.

This test uses the same batch, the same specifications, and the same grade of plates to carry out the material properties test, which is used to design beams and columns, respectively. According to the provisions of GB/T228.1–2010 [24], standard tensile material specimens are processed. As shown in Figure 3, the ordinary steel plate specimen is a plate, and the bolt sample is a bar. Table 2 shows the test results of each specimen material.

### 2.3. Experimental Test Setup and Loading Procedure

**Figure 4:**
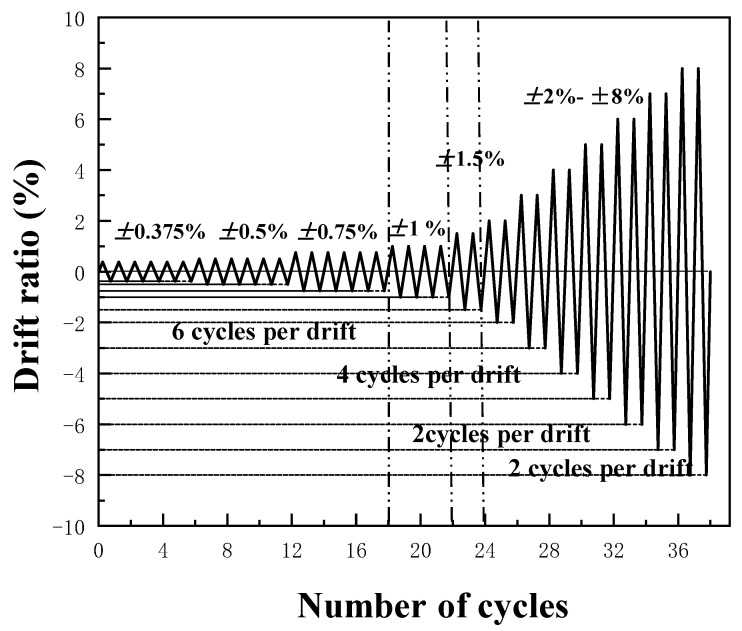
Loading protocol.

**Figure 5:**
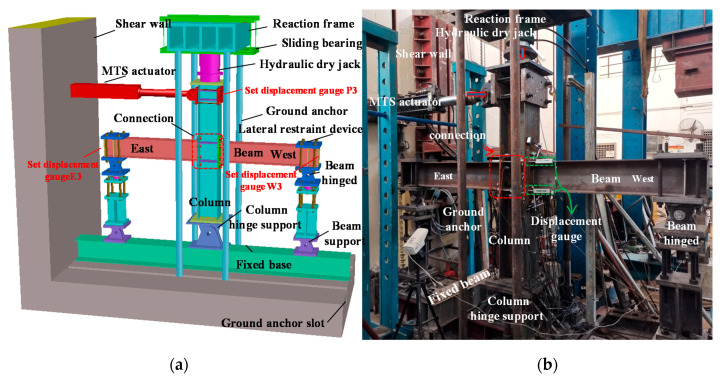
Test setup: (**a**) Schematic of test setup; (**b**) Laboratory test setup.

The experiment was conducted on the MTS hydraulic servo loading system of the State Key Laboratory of Subtropical Building Science of the South China University of Technology, Guangzhou, China. The test loading device of the intermediate column joints is illustrated in Figure 4, which includes beams, columns, connections, MTS actuators, lateral supports, loading jacks, and hinged supports. The top of the column is connected to the sliding support via a ground anchor, while the bottom end of the column is connected to hinged support via the fixed base. Each beam tip adopts a hinged boundary condition, and a lateral fixture restraint device is used to prevent out-of-plane torsional instability. The force of column axis is applied from the top of the column by a hydraulic jack. In order to simulate seismic loads, a hydraulic servo actuator with a range of 600 kN was used to apply periodic loads to the top-side of the column. The whole process of the cyclic load is controlled by the layer angular displacement. The layer angular displacement is the height of the column multiplied by the layer displacement angle; that is, *D_dr_ = H × θ_dr_*, where *H* is the column height. During the test, the loading speed of the elastic section is 0.5 mm/min. After the specimen enters plastic, the loading speed increases to 1.2 mm/min. Stop the cyclic loading until the beam tip load drops to 40% of the peak load or when the weld or bolt of the joint specimen is broken. The loading system adopts the loading procedure for cycle testing proposed by SAC Joint Venture (1997) [25]. The loading amplitude is shown in Figure 5, and the specific parameters are summarized in Table 3.

### 2.4. Measuring Point Arrangement

**Figure 6:**
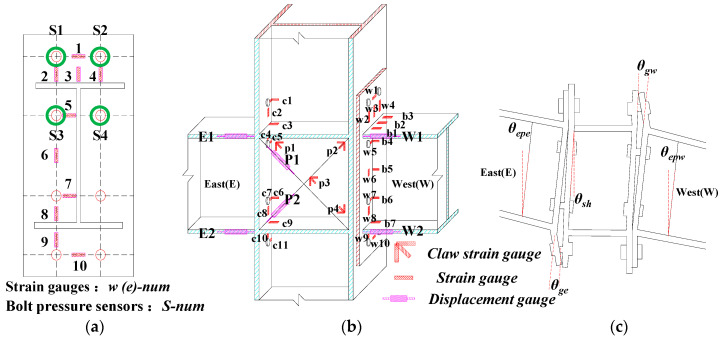
Schematic diagram of measuring point’s layout and rotation angle (*θ*) definition: (**a**) End-plate strain gauge arrangement; (**b**) Strain gauge test setup; (**c**) Schematic of the relative rotation angle of the beam column.

The displacement and strain measurement points of the intermediate column joints are symmetrically laid, as illuminated in Figure 6. The east and west beams of the intermediate column joint in the figure are denoted by “E” and “W”, respectively. Four displacement gauges are arranged along the centerline of the upper and lower flanges of the beam to measure the relative deformation of the end-plate and the column flange, corresponding to the numbers E1, E2 and W1, W2, and two pull on the rope displacement gauges that are arranged diagonally along with the panel zone. The shear deformation of the column web is measured, corresponding to the numbers P1 and P2; at the same time, horizontal displacement gauges are arranged at the end of the east and west beams and the top-side of the column, and the corresponding numbers are E3, W3, and P3 to measure the beam–column joints horizontal displacement.

As seen in Figure 6a, 20 strain gauge measuring points are arranged on the end-plates, which are located on the end-plates of the east beam and west beam and are numbered e1–e10 and w1–w10, respectively; as shown in Figure 6b, they are only on the flange and web of the west beam. These plates are equipped with 7 strain gauge measuring points, corresponding to the numbers b1–b7; 11 strain gauge measuring points are arranged on the column web and flange, corresponding to the numbers c1–c11, respectively, for monitoring each component of the joint development of stress. The four claw strain gauge measuring points arranged in the panel zone are used to monitor the shear buckling, which are respectively numbered p1–p4. Bolt pressure sensors are respectively arranged on the two rows of bolts on the end-plate, and the corresponding numbers are S1–S4. Since the edge column joint is only connected to the end-plate by the one-sided column flange, except for the other side’s test device, displacement, and strain measurement points, the rest are the same as the intermediate column joints. The above data signals are collected by the DH-3816N (DongHuaTest, Taizhou, China) static strain measurement system for the strain and displacement of each measuring point. The test load and displacement of the loading point on the beam tip or the top-side of the column are automatically recorded and collected by the inherent built-in force and displacement sensors of the MTS actuator, respectively.

### 2.5. Measurement of Rotation Angle (θ)

**Figure 7:**
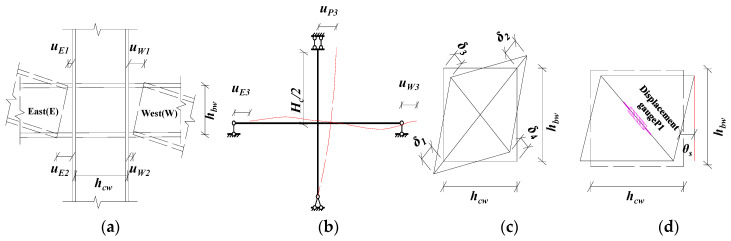
Method for measuring rotation angle of beam-to-column joints: (**a**) Method 1 calculating the angle of the beam end-plate; (**b**) Method 2 calculating the angle of beam end-plate; (**c**) Method 1 calculating the shear angle; (**d**) Method 2 calculating the shear angle.

The displacement measured by the gauge set in the test includes the overall rigid body rotation of the joint specimen, the rotation of the end-plate, and the deformation of the panel zone. Figure 7 is a schematic diagram of the relative rotation angle of the beam and column. Based on the component method, the rotation angle *θ_g_* of the end-plate connecting the beam–column joint includes the relative rotation angle *θ_ep_* [26] between the end-plate and the column flange and the shear rotation angle *θs* of the panel zone. Furthermore, the shear rotation angle and relative rotation angle between the column flange and beam can be derived from Equations (1)–(5), respectively.

- Relative rotation angle between the end-plate and column flange *θ_ep_*:

Method 1: The horizontal displacement difference between the upper and lower flanges of the beam is used to calculate *θ_ep_*.

East side beam:

(1a) $$\theta_{epe}=\frac{u_{E1}-u_{E2}}{h_{bw}}$$

West side beam:

(1b) $$\theta_{epw}=\frac{u_{W1}-u_{W2}}{h_{bw}}$$

Method 2: Calculate *θ_ep_* using the displacement difference between the column top and beam tip.

East side beam:

(2a) $$\theta_{epe}=\frac{u_{P3}+u_{E3}}{H_{c}/2}$$

West side beam:

(2b) $$\theta_{epw}=\frac{u_{P3}-u_{W3}}{H_{c}/2}$$

- Shear angle of panel zone *θ_s_*:

Method 1: [27] Method to calculate shear deformation.

(3a) $$\Delta =\left(\delta_{1}+\delta_{2}+\delta_{3}+\delta_{4}\right)/2=\left(u_{P1}-u_{P2}\right)/2$$

(3b) $$\theta_{s}=\Delta \sqrt{h_{bw}^{2}+h_{cw}^{2}}/\left(h_{bw}h_{cw}\right)=\left(u_{P1}-u_{P2}\right)\sqrt{h_{bw}^{2}+h_{cw}^{2}}/\left(2h_{bw}h_{cw}\right)$$

Method 2: Cosine method to calculate the shear angle.

(4) $$\theta_{s}=\frac{\pi }{2}-\arccos\left[\frac{(h_{bw}^{2}+h_{cw}^{2})-{\left(\sqrt{h_{bw}^{2}+h_{cw}^{2}}+u_{P1}\right)}^{2}}{2h_{bw}h_{cw}}\right]$$

- The relative rotation angle of the beam–column joint *θ_g_*:
  (5a) $$\theta_{ge}=\theta_{epe}+\theta_{s}$$
  (5b) $$\theta_{gw}=\theta_{epw}+\theta_{s}$$
  where *H_C_* is the column height. *h_bw_* and *h_cw_* are the height and width of the panel zone, respectively, corresponding to the spacing between the beam and the centerline of the upper and lower flanges of the column, and the letter *u* corresponds to the values of the displacement gauges. Through the on-site calibration of the test, the results of the two measurement methods of data analysis, Method 1 and Method 2, can be verified with each other. The overall results are consistent, but there is a scope of application. For example, when the joint is in the elastic stage, the rotation angle of the joints calculated by Equation (2) for calculating *θ_ep_* is accurate. After the connection enters plasticity, the shear angle of the joints panel zone accounts for 2/3 of the total angle, and the rotation of the column also accounts for a large proportion, which no longer satisfies the minor angle method, i.e., sin*θ ≠ θ ≠* tan*θ ≠* Δ/*H_C_*, making the joint angle *θ_gw_* (*θ*) calculated by Equation (5) too small. However, for the calculation of *θ_s_* in Equation (4), when the deformation value of the test is larger than the displacement measurement range used, the displacement of the displacement meter on a single diagonal edge fails, resulting in the ultimate shear deformation of the panel zone that cannot be directly measured accurately. This makes it difficult to accurately reflect the joint’s moment–angle curve from subsequent calculations. In the follow-up study of this paper, the relative rotation angle of the end-plate (*θ_ep_*) and the shear rotation angle (*θ_s_*) are both adopted by Method 1.

## 3. Result

### 3.1. Test Phenomenon and Failure Modes

#### 3.1.1. Specimen IC-EP1

**Figure 8:**
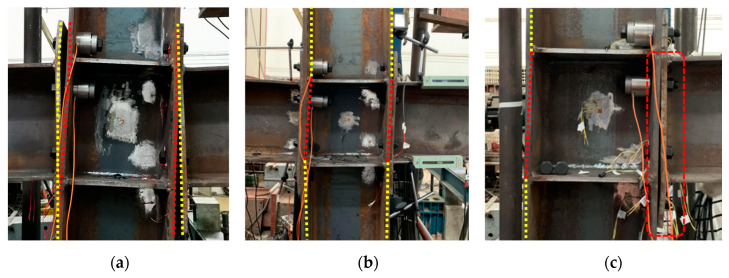
Failure modes of specimens under cyclic loading: (**a**) Specimen IC-EP1; (**b**) Specimen IC-EP2; (**c**) Specimen EC-EP1/EC-EP2.

**Figure 9:**
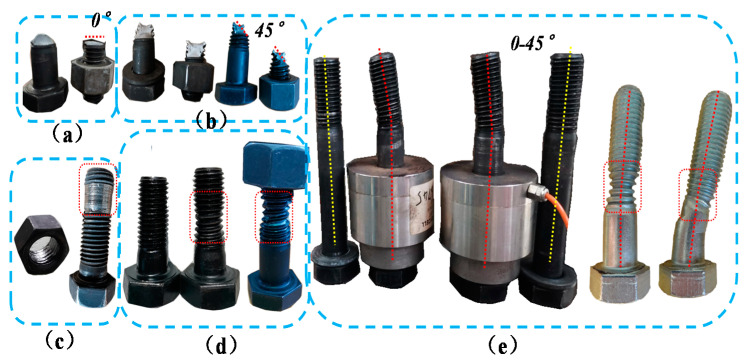
Various deformation forms of bolts: (**a**) Tensile failure; (**b**) Bolt oblique shear failure; (**c**) Bolt thread worn failure; (**d**) Bolt shank necking failure; (**e**) Bolt shank axis bending and necking.

For IC-EP1 specimens, at the initial stage of loading, the relationship between load and displacement is basically linear. As the amplitude load of the layer angular displacement increases, we observe the gap between the end-plate and the column flange surface also reciprocating change. In the process of increasing the gap, due to the alternating positive and negative bending moments, the center of rotation also changes at the center points of the upper and lower flanges of the beam, which causes the end-plate and the lower flange of the beam to yield first. Then, the upper flange of the beam yields, and the beam–column yields later. For cyclic loading to 126 mm, the corresponding layer displacement angle is 0.06 rad, the load limit state is reached, the weld between the end-plate of the west beam and the lower flange of the beam suddenly cracked, and the connection failure mode is a brittle failure, as demonstrated in Figure 8a. The upper row of bolts on the east side is broken. When the bolt fails, the angle of the failure surface of the bolt shank is 0° tensile failure, while the second row of bolts appears necking, as shown in Figure 9a,d, corresponding to failure mode I. The end-plate is also separated from the column flange, while the top two rows of bolts bear the greatest tensile force; these bolts are broken or necked. When the upper bolts suddenly break, the sharp unloading of the connection causes the bolt axes of the lower two rows to bend, as displayed in Figure 9e. The whole process of panel zone shear deformation is not obvious.

#### 3.1.2. Specimen IC-EP2

**Figure 10:**
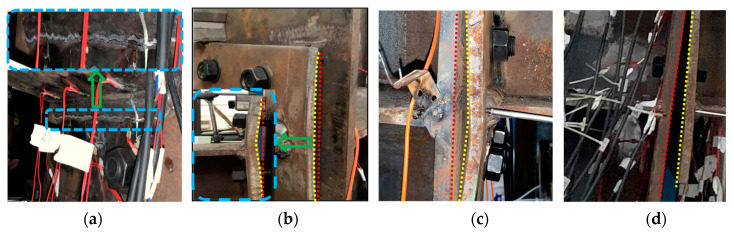
Local deformation of specimens: (**a**) Weld fracture; (**b**) Upper flange end-plate bending; (**c**) Lower flange end-plate bending; (**d**) Separation of end-plate and column web.

For the IC-EP2 specimen, during the first three drift cycles, the test phenomenon is similar to the IC-EP1 specimen. The column web reaches the buckling strength at the rotation angle of 0.0047 rad. During the later loading period, the end-plates near the bolt holes gradually yielded, and both the upper and lower flanges showed bending deformation, as shown in Figure 10b,c. The top-side of the column continues to be loaded to 146 mm, and the failure mode is excessive shear deformation of the panel zone. As shown in Figure 8b, when the shear deformation of the sample is too large, the loading stops, and the connection failure mode is obvious shear buckling. The upper and lower rows of bolts on the east and west side beam both suffer from oblique shear failure and bolts shank axis bending and necking, corresponding to failure modes II and III, as shown in Figure 9b,e.

#### 3.1.3. Specimens EC-EP1 and EC-EP2

For the edge column EC-EP1 and EC-EP2 specimens, the displacement is loaded to 100 mm, and the failure mode appears as shear deformation and end-plate bending. As displayed in Figure 8c, compared to the shear angle of the IC-EP2 specimen when the specimen was a failure, their shear angle was 0.04 rad, and the shear angle of the specimens EC-EP1 and EC-EP2 were 0.76 and 0.54 times that of IC-EP2, respectively. The maximum moment and shear of the panel zone is about 60% of that of the intermediate column joint, and the connection failure mode involves a large bending of the end-plate and slight shear deformation of the panel zone. Under cyclic loading, the edge columns only bear shear and moments on one-sided connections, and the panel zone incurs shear deformation, which makes the bolt shank axis at the connection tilt horizontally and squeeze the bolt hole wall. Under the reciprocating load, the bolts thread is ground, and the sliding tooth is damaged. The bolt failure modes are the necking failure of the upper and lower rows of bolts and the failure of the thread of the worn teeth of the middle two rows of bolts, as shown in Figure 9c,d.

The failure modes of the bolts in the specimens may affect the performance of the connections to a large extent. Moreover, the influence of connection parameters on the performance of the connections is related to the failure modes. Therefore, it is necessary to observe different typical failure modes of the bolts. There are three typical failure modes for bolts. When the bolts fail, they can be roughly divided into failure mode I, failure mode II, and failure mode III, according to the angle of the failure surface of the bolt shank. The corresponding included angles are 0°, 0–45°, and 45°. Table 4 gives a comparison of typical failure modes in four rows of bolts for different test specimens.

## 4. Discussion

### 4.1. Moment–Rotation Hysteresis Curves

For the cyclic loading of the test specimen, the shape of the hysteretic bending moment–rotation curve is roughly similar before the first three drift ratios. For the cyclic loading of all specimens, the shape of the hysteretic bending moment–rotation curve is generally similar. The moment–rotation function relationship can be considered to be roughly linear before the rotation angle is 0.005 rad. In the inelastic stage, with the increase of the layer angular displacement, the moment–rotation curve turns to the *x*-axis, and the connection rotation stiffness gradually degraded. Under cyclic loading and reciprocating action, the flexural resistance of the connection also decreases. The plastic deformation ability of the four test specimens is favorable; the limit rotation angle is greater than 0.03 rad required by the US FEMA [28], and the maximum rotation of the test specimen IC-EP2 reaches 0.07 rad.

**Figure 11:**
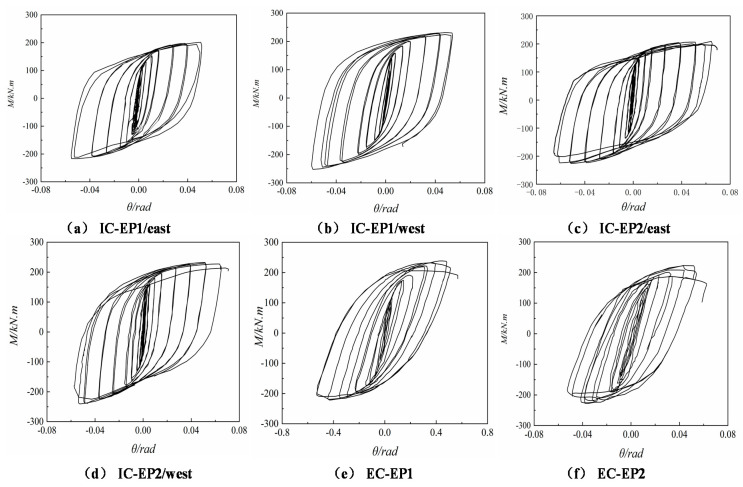
Moment (*M*)–rotation (*θ*) hysteresis curves. (**a**) East connection of specimen IC-EP1; (**b**) West connection of specimen IC-EP1; (**c**) East connection of specimen IC-EP2; (**d**) West connection of specimen IC-EP2; (**e**) Specimen EC-EP1; (**f**) Specimen EC-EP2.

Until the drift ratio reached 4%, the test specimens with the extended end-plate exhibited stable and reliable hysteresis behavior with only small strength degradation. However, the stiffness is severely degraded. It can be seen from Figure 11 that the bolt diameter of the test specimen ICEP1 is smaller than that of the test piece ICEP2, and its failure modes are bolt fracture and weld cracking. Plastic hinges are not formed on the beam ends of all specimens, so the hysteretic curve has no distinct yielding platform, which is stable and plump, indicating that the joint energy dissipation is satisfactory, the components in the connection did not slip during the test, and the joints are not cracked before the same level of drift ratio. The ductility performance is very obvious; it is an ideal seismic dissipation mode in general. Therefore, it can be applied in the practical design of this type of connection.

### 4.2. M–θ Envelope Curves

**Figure 12:**
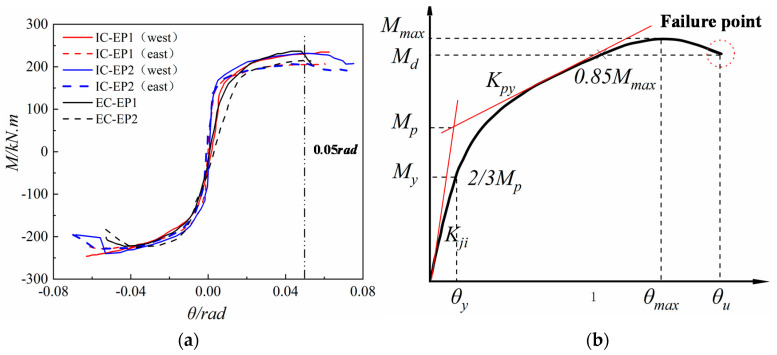
Moment (*M*)–rotation (*θ*) envelope curves and key parameters definition of joints: (**a**) Skeleton curve; (**b**) Key parameters definition in the moment–rotation curve of connections.

The key parameters of the joint’s moment–rotation relationship are defined in Figure 12b. According to the definition method of yield moment in reference [29,30], *M_max_* is the maximum moment value (peak moment) experienced by the tested specimens during loading, and the corresponding beam–column relative rotation angle is *θ_max_*. At the same time, the failure moment *M_d_* = 0.85*M_max_* is defined, and *M_d_* corresponds to the limit rotation angle *θ_u_*. The joint bending moment–rotational skeleton curve of each specimen is shown in Figure 12a, while the corresponding parameters of the load characteristic points are shown displayed in Table 5. The moment of the joint is the product of the load value *F* at the beam tip and the distance *L*_0_ from the load point to the surface of the column flange. When the four specimens reach 0.05 rad, the joint can still endure more than 0.85 *M_max_*. According to the failure mode of the extended end-plate joint under cyclic loading, it can be considered that all joints still have certain robustness.

By comparing the moment–rotation skeleton curve shown in Figure 12a, it can be clearly seen that the initial rotational stiffness of the intermediate column is much greater than that of the edge column joints, but the ductility of the intermediate column joint is reduced by approximately 15%. In addition, compared with the specimens ICEP1 and ICEP2, the flexural resistance capacity and rotation capacity of the specimens ECEP1 and ECEP2 are slightly lower, because the edge column joints have only a one-sided end-plate to limit the relative rotation of the panel zone, while the change of the boundary conditions of the panel zone reduces the flexural resistance capacity and rotation capacity by 9.8% and 8.3%, respectively. Meanwhile, the ECEP2 end-plate thickness of the specimen is 20 mm. Compared with the specimen ECEP1, the flexural resistance capacity and the rotation capacity are increased by about 5.8% and 6%, and the initial rotation stiffness is increased by 7.4%. Compared with ICEP2, the bolt diameter of the specimen ICEP1 is increased by 4 mm, and the flexural resistance capacity, rotation capacity, and initial rotation stiffness change little, but this causes the failure mode to change from the weld crack at the end-plate to the excessive shear deformation of the panel zone. These results show that the mechanical behaviors of the east–west connection of the intermediate column joint are basically the same. Compared with the edge columns, the intermediate column joint has significantly improved initial rotational stiffness and rotational capacity, while the flexural resistance capacity remains unchanged.

### 4.3. Energy Dissipation

**Figure 13:**
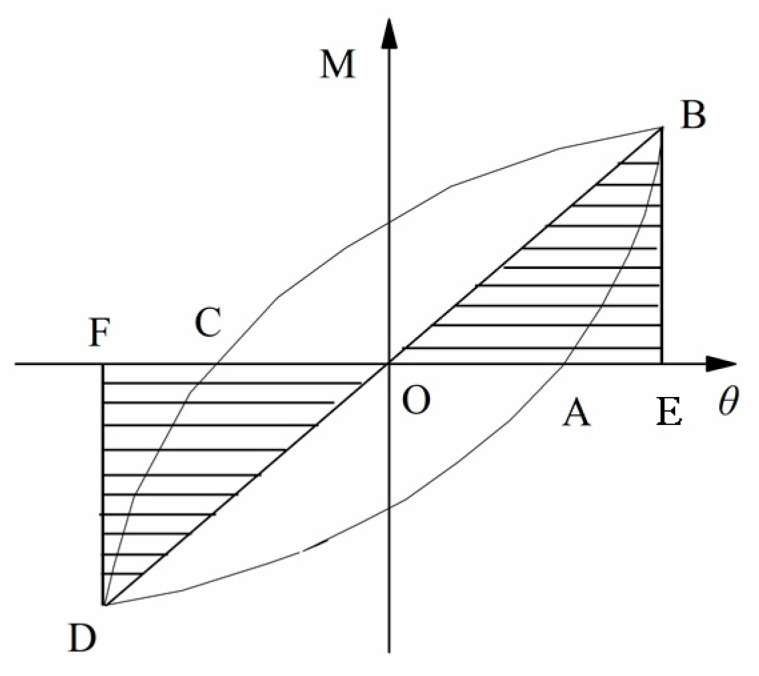
Definition of a hysteretic loop.

Three key parameters are adopted to characterize the loop energy dissipation capacity of the beam–column joints, which are namely the total dissipated energy (*W_total_*), equivalent viscous damping coefficient (*ξ_e_*), and energy efficiency factor (*E_e_*). Total dissipated energy (*W_total_*) is the cumulative dissipated energy (*W*) described as the function of the joint rotation angle *θ*, where W is the area of the *M–θ* hysteresis curve at a specific cycle. The energy dissipation capacity (*E_e_*) and the equivalent viscous damping coefficient (*ξ_e_*) can be calculated according to the formula specifically expressed as Equations (6) and (7). The energy dissipation coefficient *E_e_* is defined as the ratio of the total energy of the component in a hysteretic loop to the elastic energy of the component. *E_e_* represents the ratio of the occupied elastic energy to the total energy of the structure in a closed hysteresis loop. The larger the *E_e_*, the stronger the energy dissipation ability of the tested connection. In Figure 13, where SABC and SCDA refer to the upper half area and lower half area of the hysteresis loop, respectively, and SOBE and SODF refer to the corresponding triangular areas. The parameter ∆ is defined as the relative difference between the total energy consumption of each specimen and the control specimen EC-EP1, which is used for the comparison and analysis of each sample and the control sample EC-EP1. The energy consumption index results are summarized in Table 6.

(6) $$E_{e}=\frac{S_{ABC}+S_{CDA}}{S_{OBE}+S_{ODF}}$$

(7) $$\xi_{c}=\frac{E_{e}}{2\pi }$$

(8) $$\Delta =\frac{\left|W_{total}{}_{\left(i\right)}-W_{total}{}_{\left(EC-EP1\right)}\right|}{W_{total}{}_{\left(EC-EP1\right)}}\times 100\%$$

**Figure 14:**
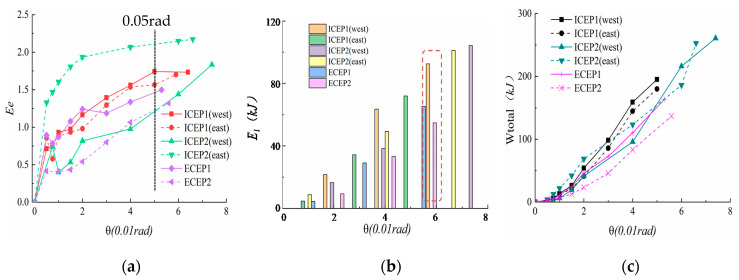
Energy dissipation: (**a**) Equivalent viscous damping; (**b**) Energy dissipated during in each step; (**c**) Accumulated energy dissipation.

Table 6 compares the energy dissipation capacity (*E_e_*), equivalent viscous damping coefficient (*ξ_e_*), and total energy dissipation (*W_total_*) in the limit state. Figure 14 exhibits the equivalent viscous damping coefficient (*ξ_e_*) of each hysteresis loop versus the drift ratio and the total dissipation energy (*W_total_*) related to the drift ratio. These test results show the following:

(1) As shown in Figure 14a, as the cyclic displacement increases, the *E_e_* values of the four specimens change. Under the relative rotation angle of the joints of 0.05 rad, the *E_e_* value of the loaded specimens in all experiments is greater than 1, which indicates that all specimens have acceptable energy dissipation capacity.
(2) Table 6 shows that the energy dissipation coefficient of the IC-EP2 specimen exceeds 2.2 when the specimen is a failure, indicating that this type of joint is an ideal seismic energy dissipation connection. The IC-EP1 and EC-EP1 specimens with the same parameter configuration are only the difference between the single and double-sided end-plates, which increases the total energy consumption of the east and west sides of the IC-EP1 specimen by 30.7% and 41.6%. For the setting of joints, it indicates that for the edge column and the intermediate column, the energy dissipation capacity of the edge columns should be used as the reference value for the lower limit of the design in the seismic energy dissipation design.
(3) Compared with the IC-EP1 and IC-EP2 specimens, the use of bolts with a smaller diameter makes the connection fail prematurely, as displayed in Figure 14c, and the corresponding joint energy consumption is also reduced by about 21%.
(4) As shown in Figure 14b,c, the energy dissipated in each step (*E_i_*) and the cumulative energy dissipation (*W_total_*) are constantly increased. At a relative rotation angle of 0.05 rad, the *E_i_* of the four specimens are basically more than 60 kJ, and the cumulative energy dissipation of the intermediate column specimens during failure varies from 170 to 260 kJ, showing that the energy consumption effect of the structure in the early stage is very distinct. The two specimens EC-EP1 and EC-EP2 showed similar responses in the limit state, but the EC-EP2 specimen increased the thickness of the end-plate by 4 mm, corresponding to a 14.5% decrease in total energy consumption.

### 4.4. Rotation Ability and Ductility

The test results in Table 5 show that the ductility coefficient *μ_θ_* of the connection joint of the extended end-plate varies from 7.8 to 10.6. The test specimens show favorable ductility. Regarding the intermediate column joints of specimens IC-EP1 and IC-EP2, due to the symmetry of the loading method and the setting of boundary conditions, the initial rotational stiffness, flexural resistance strength, and ductility of the beam connection joints on the east and west sides of the specimen are basically the same, and the error range is controlled within 5%. This shows that the mechanical behavior of the two sides of the intermediate column is basically the same under the action of the same positive and negative displacement values. However, when the M20 bolt was replaced with the M24 bolt in the intermediate column test, the initial rotational stiffness and flexural resistance of the joint increased by 14.6% and 6.3%, respectively, while the corresponding rotational capacity decreased by 3.29%. The difference between the specimens IC-EP1 and EC-EP1 is only on the single and double sides of the column connection; here, the initial rotation stiffness of the joints differs by 42.6% and the flexural resistance capacity differs by 5.8%, indicating that the connection between the edge column and the intermediate column joints on both sides has an effect on the initial rotational stiffness. Compared with EC-EP1, the end-plate thickness of specimen EC-EP2 increased by 4 mm, and the other values only changed slightly, while the corresponding ductility coefficient decreased by 7.5%. These results show that the thick end-plate joints correspond to higher stiffness, and the ductility coefficient decreases with the increase of the end-plate thickness, because the end-plate bending component restricts the rotation of the joint. It also slows down the decrease of the moment of the joint, resulting in a higher strength, stiffness, and lower ductility of the joint. In the case of intermediate column joints, larger bolt diameters, thicker end-plates, and weaker steel beams should be used to show satisfactory ductility and seismic performance, as well as ensure higher flexural resistance strength to avoid bolts’ premature brittle fracture, and give full play to the deformation performance of end-plates and steel beams. This is in line with the design concept of “strong joint and weak member”.

The Chinese code GB50011-2010 [31] for multi-story high-rise steel structures specifies an elastic layer angular displacement [*θ_e_*] of 1/250 rad and an elastic–plastic layer angular displacement [*θ_p_*] of 1/50 rad. As shown in Table 5, the yield connection rotation (*θ_y_*) is approximately 1.18–1.98 times [*θ_e_*], and the failure connection rotation (*θ_u_*) is 2.5 to 3.5 times [*θ_p_*]. To satisfy the ductility requirement in seismic design, FEMA 350 [28] suggests a ductility limit of 0.03 rad. It is indicated by comparison that the extended end-plate connections show admirable rotation ability, satisfying the specified earthquake design requirements in both GB50011-2010 and FEMA 350. The ductility coefficient of the EC-EP1 edge column end-plate connection is 10.6; on the other side, the end-plate connection is added to become the IC-EP1 test specimen. The ductility coefficient drops to 9.8, indicating that the edge column rotation ability is better than the intermediate column joint. Increasing the bolt diameter to 24 mm, the ductility coefficient of the IC-EP2 specimen decreased to a lower level.

Refer to [32]’s method, which is addressed to the definition stiffness ratio for the joints. Meanwhile, the stiffness ratio function (*K_i_*/*K*) can be used to indicate the deterioration of the joint stiffness with the increase of the number of cycles, where *K_i_* is the secant stiffness of the joint in the considered cycle, and *K* is the initial rotational stiffness. The secant stiffness *K_i_* is defined by Equation (9).

(9) $$K_{i}=\frac{M_{k}-M_{l}}{\theta_{k}-\theta_{l}}$$

**Figure 15:**
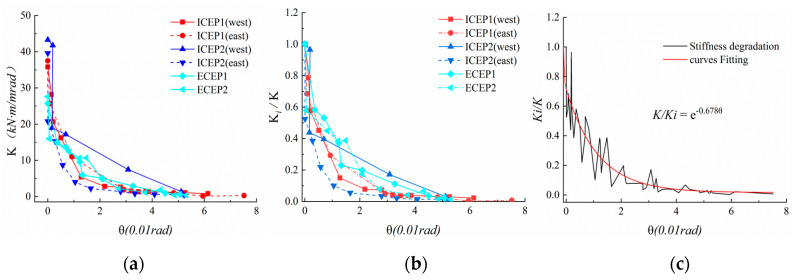
Stiffness degradation curves: (**a**) Stiffness value degradation; (**b**) Stiffness degradation ratio; (**c**) Stiffness degradation fitting curve.

The degradation of stiffness is delineated in Figure 15. In the variation law of the stiffness degradation curve, similar trends of four specimens were found. In the elastic phase, the joint stiffness basically remained in the range from 25.7 to 63.3 kN·m/mrad, and when the rotation reached 0.005 rad, the joint entered the plastic phase. As shown in Figure 15c, the joint stiffness begins to degenerate exponentially. Figure 15a,b shows the specimen EC-EP2. The stiffness degradation is slower than that of the other three specimens. Perhaps because the bolt diameter is equal to the end-plate thickness, the mechanical behaviors of the joint components are well-coordinated, and the slowing effect of the stiffness degradation regular can be roughly evaluated: Specimen EC-EP2 > Specimen EC-EP1 > Specimen IC-EP1 > Specimen IC-EP2. When the rotation is 0.05 rad, most of the components of the specimen joint enter the plastic stage, and the joint stiffness is less than 0.1 times the initial rotational stiffness.

### 4.5. Classification of the Tested Connections

According to the rotation stiffness and strength of the moment–rotation envelope curve, it is determined whether the connection joint of the end-plate of the intermediate column and edge column belongs to a semi-rigid, partially equal strength connection. The classification method in reference to the European standard EN1993-1-8 is as follows:

1. Classification according to stiffness: when *K_ji_ ≥* 8*EI_b_/L_b_* (braced frame, *EI_b_/L_b_* is the linear stiffness of the beam, where *E*, *I_b_* and *L_b_* are the elasticity modulus, second moment of area, and the length of the steel beam, respectively) or *K_ji_ ≥* 25*EI_b_/L_b_* (non-braced frame), it is a rigid connection; *K_ji_* ≤ 0.5*EI_b_/L_b_*, it is nominally pinned; 0.5*EI_b_/L_b_* < *K_ji_* < 8*EI_b_/L_b_* (braced frame) or 0.5*EI_b_/L_b_* < *K_ji_* < 25*EI_b_/L_b_* (non-braced frame), it is a semi-rigid connection.
2. Classification according to strength: when *M_d_ ≥ M_bp_* (where *M_bp_* represents the design plastic flexural resistance of the steel beam), it is a full-strength connection; when 0.25Mbp < Md < Mbp, it is a partial-strength connection; when 0.25Mbp > Md, it is a nominally pinned connection.

**Figure 16:**
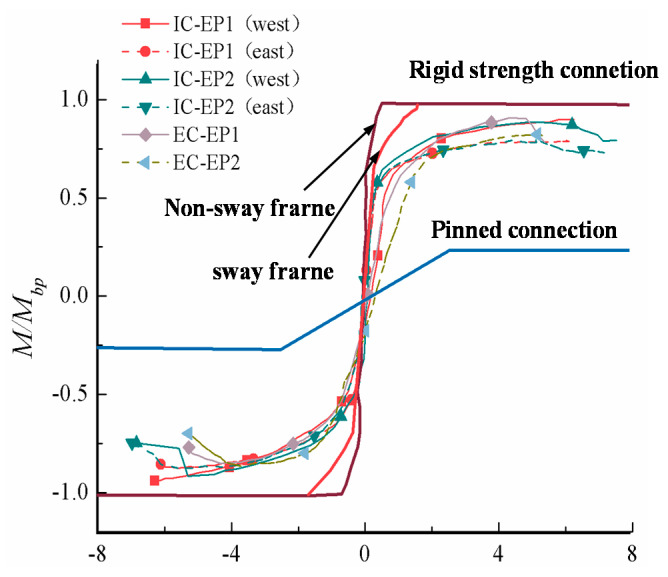
Classification of tested connections.

The *M_bp_* of the beam section used in the test is 261.3 kN·m. The peak bending moments of the four test specimens in Figure 16 are 93.8%, 89.6%, 90.5%, and 86.4% of *M_bp_*, respectively. Table 5 shows that the initial rotational stiffness of all test specimens is between 0.5 *EI_b_/L_b_* (7.9 kN·m/rad) and 8EIb/Lb (127.2 kN·m/rad). The classification of tested connections is illustrated in Figure 16, the extended end-plate joints in the test are divided into semi-rigid connections according to the stiffness; the flexural resistance of the all specimens are (0.86–0.94) *M_bp_*. They are classified as partial-strength and semi-rigid joints.

## 5. Finite Element Verification and Exploratory Numerical Research

### 5.1. Finite Element Modeling

#### 5.1.1. Material Models

**Figure 17:**
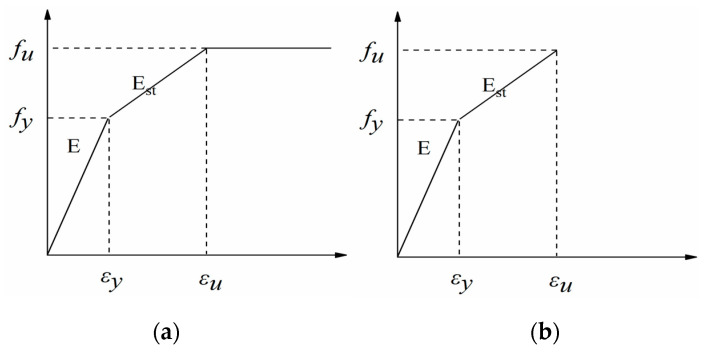
Material constitutive model: (**a**) Steel constitutive model; (**b**) Bolt constitutive model.

The numerical analysis builds an efficient and precise model on the ABAQUS/Standard^®^ [33] module to simulate the mechanical behavior of the test joint specimen. All parts are modeled using the 8-node linear brick incompatible mode element (C3D8R) to emulate the connections, as shown in Figure 17. The stress–strain relationship of the steel can be simplified to a multi-linear relationship, and it considers the plastic hardening of the material. The Von Mises yield criterion is adopted to determine whether the steel reaches the yield point in the multi-axial stress state. A bilinear kinematic hardening model was applied to the high-strength bolt constitutive model, which is usually applied for high-strength steel. Various finite element (FE) model material parameters and actual tensile test results correspond to the data in order to better verify the mechanical behaviors of the joint. See Table 7 for detailed information about the material properties of steel and bolts. A tie contact is used for the welding relationship between the end-plates and steel beams and does not consider another weld modeling. Normal hard contact and tangential penalty function are used to simulate the surface interaction between other parts, while hard contact was used for normal contact to simulate the extrusion phenomenon between the bolt and the plate. A penalty function was used for tangential contact to simulate friction with a coefficient of 0.44 between the end-plate and the column flange.

#### 5.1.2. Model Description

Considering the symmetry of materials and boundary conditions, while saving calculation time and reducing calculation expense, this group of finite elements uses full-model mesh, half-model mesh, and quarter-model mesh, which were built for numerical analysis; the corresponding total number of elements is about 104,000, 52,000, and 26,000 elements, respectively. The structured meshing technique was used to form a proper element shape, which can achieve the accuracy of the finite element analysis results, especially for bolts and end-plate parts. 

**Figure 18:**
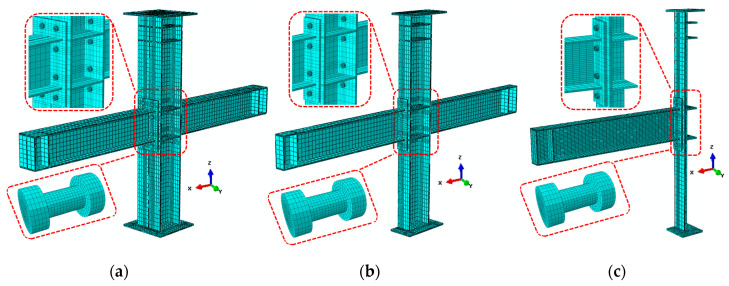
Joint model and mesh of steel part: (**a**) Full-model mesh; (**b**) Half-model mesh; (**c**) Quarter-model mesh.

The assembly and meshing of the specimen model are shown in Figure 18. Due to the difference of boundary conditions, the intermediate column joint adopts a half-model and quarter-model, and the edge column joint only adopts a half-model for simulation calculation. Each model applies three types of loads: the first was the constant load that was applied at the middle of the bolt shank to simulate the pretension force, the second is to maintain an axial compression ratio of 0.3 and apply a constant pressure value on the column top, while the third was the cyclic load that was applied in the form of small steps at the top-side of the column to produce the bending moment on the connection. It is applied to the top-side of the column in the form of cyclic small incremental displacement, and the displacement loading of the numerical simulation is consistent with the loading protocol of the experiment. According to the experimental setup, the symmetrical model only deforms in the XOZ plane, so the initial setting limits the UY translation direction and RX and RZ rotation. It can be assumed that the top of the column is sliding support, which corresponds to limiting the rotation in the RY direction; the bottom of the column is hinged and the support restricts UX and UZ translation; the beam tip is hinged support, which restricts UZ translation.

### 5.2. Validation

**Figure 19:**
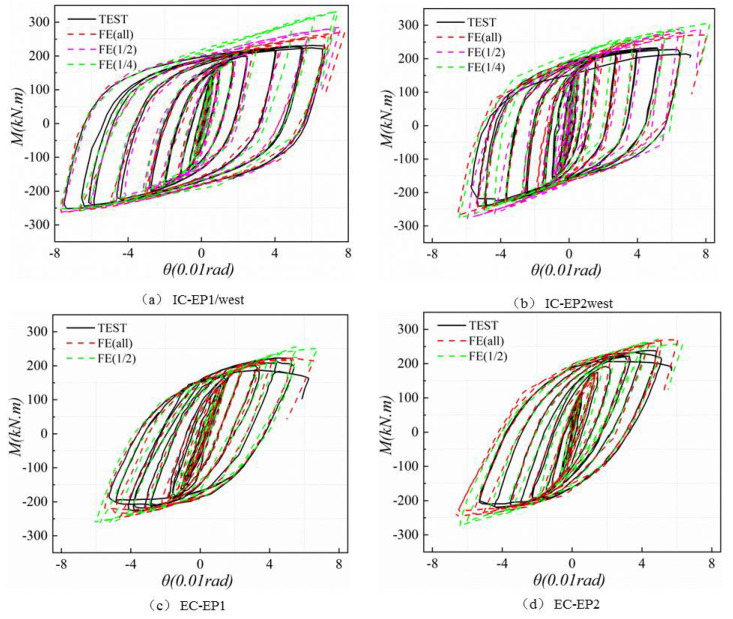
Comparison between experimental and FE results for the four tested specimens. (**a**) IC-EP1/west; (**b**) IC-EP2west; (**c**) EC-EP1; (**d**) EC-EP2.

As presented in Figure 19, for all specimens, the *M–θ* relationship at the initial elastic stage is almost linear, and the FEA of all models and the test hysteresis curves show a satisfactory agreement. With the increase of loading displacement, the constitutive relationship of steel used in the finite element is that the elastic–plastic with kinematic hardening is different from the actual material curve of the test. The initial geometric defects and the test error make the FEA and the test slightly different. However, the error range is within the controllable area, the FEA of all models and the test hysteresis curve show good agreement, and the results of finite element analysis show that the establishment of the full-model (FE all) is closest to the experimental results. However, the use of creating a full-model requires a longer calculation time and greater computer storage space. On the other hand, the material and boundary of the specimen are symmetrical, and the quarter-model for the intermediate column and the half-model for the edge column is also close to the results obtained by the test. Therefore, it can be concluded that in terms of accuracy, running time, and data storage, the intermediate column joint adopts a quarter-model (FE 1/4), and the edge column joint using a quarter-model (FE 1/4). Such a finite element analysis method can meet the requirements of numerical verification.

### 5.3. Results and Discussion

**Figure 20:**
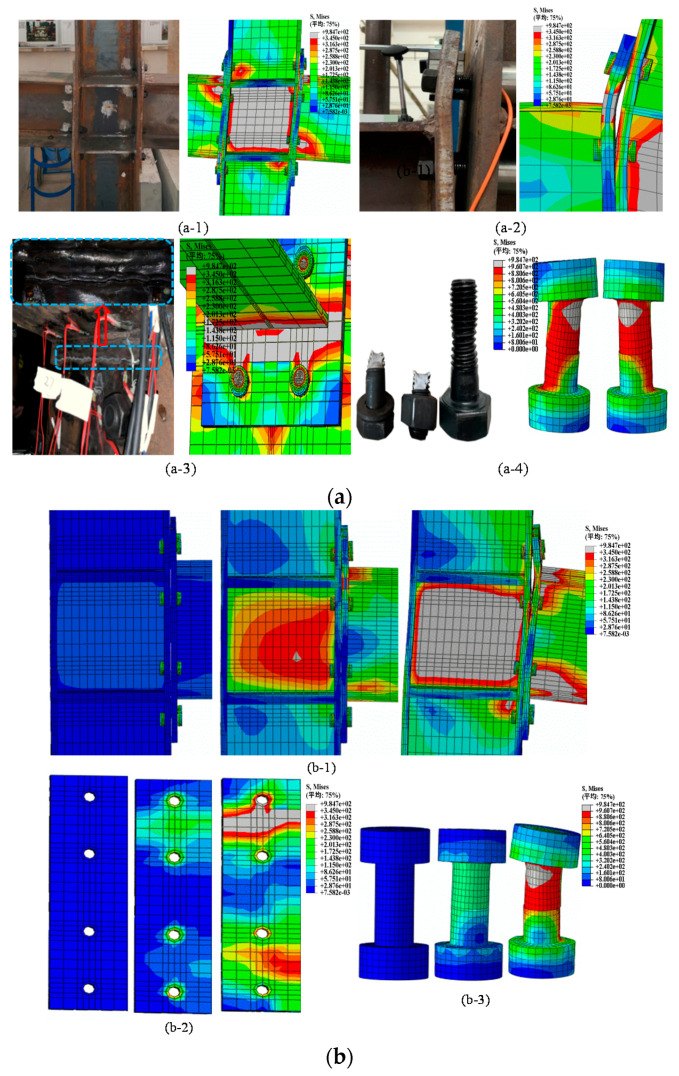
Local deformation and simulation process: (**a**) Comparison of test and finite element; (**b**) Corresponding to 0.01, 0.03, 0.05 rad joint rotation stress contour.

As illustrated in Figure 20(a-3), buckling occurs between the beam flange and end-plate of the IC-EP1 model first, and then the bolt reaches the yield stress (Figure 20(a-4)). The weakest component appears at the beam flange and end-plate. Since the end-plate is not equipped with stiffeners, the stress cannot be effectively transitioned, resulting in stress concentration. In the IC-EP2 model, the column web area has local buckling [34], as shown in Figure 20(a-1); the weakest component is the panel zone at the column web. In the EC-EP1/2 model, the end-plate incurs large bending deformation and slight shear in the panel zone of the column web, as presented in Figure 20(a-2). The weakest component is still the end-plate at the connection. For the bolt breaking phenomenon in the test, it appears as bolt shank buckling in the finite element, as displayed in Figure 20(a-4). Meanwhile, it can be seen in Figure 20b that the key components of the joint basically buckle at the rotation of 0.05 rad. Since most of the key connected components have failed, the *M–θ* skeleton curve also shows that the joint flexural resistance capacity has begun to decline, and the joint secant stiffness has degraded to a very low level.

In the test specimen and finite element model, the end-plate, column flange, column web, and bolt show similar deformation. The data of the extracted finite element show that the shear failure of the column panel zone is severely buckling. The shear rotation of the intermediate column accounts for about 2/3 of the total connection rotation. It shows that the failure mode of the connection between loading the top-side of the column or loading the beam tip is roughly similar. However, the shear deformation failure at the panel zone of the column web is quite different. In the test and the finite element, the buckling of the bottom row of bolts and the bending deformation of the column flange showed the failure mode II (Reference [35]), that is, the bolt failed, and the column web yielded.

**Figure 21:**
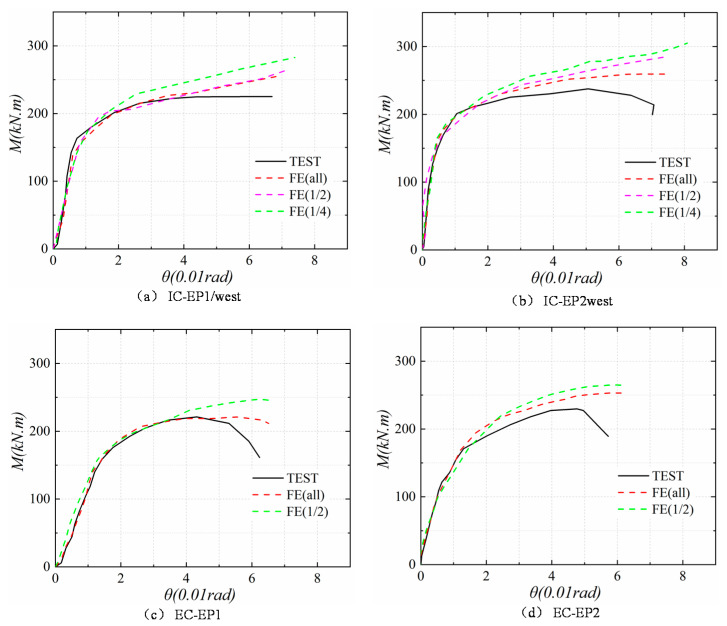
*M–θ* skeleton curves (**a**) IC-EP1 west; (**b**) IC-EP2 west; (**c**) EC-EP1; (**d**) EC-EP2.

The FEA models were validated against the experimental results. Figure 21 shows the comparison between the moment–rotation curves predicted by the FEA models and the moment–rotation curves obtained from the tests. The curves of the all specimens showed excellent agreement in the elastic range, but these had few errors in the latter stage. The steel model used in the finite element is only a single material constitutive relationship, due to the mutual coupling effect of the existence of each component of the actual structure. The finite element has difficulty fully and truly reflecting the beam–column joint constitutive relationship under such complex stress states.

In the finite element model, the calculated yield strength and initial rotational stiffness of the connection are used to define the criticality of the elastic and plastic mechanical behaviors of the joint. In the specification, the joint yield strength and the initial rotational stiffness of the joint are specified in the strain value, such as the Chinese code GB50017-2017 [23]. Using the specification high-strength bolt definition yield strength tensile residual strain 0.2% as a reference, corresponding to the initial rotational stiffness of the joint given in EC3 [19], we refer to the secant stiffness value of the moment–rotation curve at rotation 0.35% rad. Therefore, the hysteresis curve shows good consistency in some aspects of initial rotational stiffness (*k_ji_*) and joint yield moment (*M_y_*). Table 8 lists the comparison between the finite element analysis and the test results. The average and standard deviation of the ratio of the initial rotation stiffness of the finite element divided by the test are 1.003 and 0.064, respectively.

## 6. Conclusions

The following observations and conclusions can be drawn from the studies reported in this paper:

1. The bolt diameter approximately equal to the thickness of the end-plate can effectively avoid premature connection failure and excessive bending of the end plate. Loaded to 6% drift ratio, the column web basically reaches the buckling state, and the shear deformation of the panel zone is obvious. The final failure mode is the tear of the weld between the beam flange and the end-plate (WEP-BF); when the bolt diameter is smaller than the thickness of the end-plate, the failure mode is bolt break (BF), and then the end-plate and the column web are separated, causing brittle failure of the joint. More importantly, three typical failure modes are proposed for the bolts at the connection. According to the bolts, the angle of the failure surface is divided into failure modes I, II, and III, and the bolt failure modes are related to its stress state.
2. The observation results show that the hysteresis curve of the extended end-plate connection is plum and that the intermediate column joints have better rotational stiffness and rotation ability than the edge column joints. As far as the intermediate column joints are concerned, the joint stiffness is more than 80% greater than that of the edge column joints. However, the ultimate flexural resistance capacity of both is not much different, and the difference in the mechanical behaviors of the two sides’ connection of the intermediate column joints is slight. According to the EC3 classification, the joints under this loading method are still semi-rigid and partially equal-strength connection joints; the energy dissipation coefficients of the specimen joints are all over 1.3, which is the ideal seismic energy dissipation joint. During the whole loading process, the joint stiffness degrades exponentially; at a rotation angle of 0.05 rad, all components of the specimen basically enter the plastic state, and the joint stiffness is less than 0.1 times the initial rotational stiffness.
3. The column top-side loading scheme is different from the previous beam tip loading method, and the plastic hinge is also transferred from the beam end to the panel zone of the column web, which manifests itself in the form of excessive shear. This is consistent with the failure mode of some joints during the earthquake. For the comparison of the two loading methods, the joints are in the elastic stage, and the mechanical indicators such as the initial rotational stiffness are basically the same. However, after the joints enter the plastic stage, the different force transmission mechanisms result in the failure model and the ultimate flexural resistance being different.
4. The ductility coefficient *μ_θ_* of the extended end-plate joints is in the range of 9.4 to 16.1, the yield angle *θ_y_* is about (0.78 to 1.6) [*θ_e_*], and the plastic limit rotation angle *θ_u_* of the joint is about (2.5 to 3.5) [*θ_p_*]. The analysis results with the elastic layer angular displacement limit and the elastic–plastic layer angular displacement limit of the steel structure show that such joints have good ductility and meet the requirements of seismic design.
5. Use the ABAQUS/Standard module to establish the finite element full-model, symmetric half-model, and quarter-model of the connected joints. These FE models were used to cross-validate the test results. The results show that the half-model and the quarter-model can be used to simulate the intermediate column and edge column joints’ connection behavior, respectively. More importantly, FE can accurately predict the rotational stiffness of the joints in the elastic stage with sufficient accuracy. An effective simulation is given for the location where the failure occurs in the test. The results of finite element analysis are in agreement with the test, which demonstrated the reliability of the experimental results.

## Figures and Tables

**Table 1:** Configuration details of joint specimens.

Joints Type	Specimen	End-Plate Thickness (mm)	Bolt Diameter (mm)	Initial Tightening Torque (N·m)	Final Tightening Torque (N·m)	Bolt Grade	*F_pre_* (kN)	*F_c_* (kN)
Intermediate column joint	IC-EP1	16	20	280	446	10.9	155	1211
	IC-EP2	16	24	400	760	10.9	225	1211
Edge column joint	EC-EP1	16	20	280	446	10.9	155	1211
	EC-EP2	20	20	280	446	10.9	155	1211

**Table 2:** Material properties of specimens.

Sample	*t* or *d* (mm)	*E*/GPa	*f_y_*/MPa	*f_u_*/MPa	*ε_y_*/%	*ε_u_*/%	*A*/%
End-plate 1	16	210.21	366.2	541.4	0.357	50.9	27.6
End-plate 2	20	212.65	364.3	537.6	0.509	53.6	25.5
Column web	10	207.59	385.1	540.8	0. 378	28.4	27.6
Column flange	15	205.78	371.7	557.4	0.351	52.4	19.6
Beam web	8	199.47	390.5	580.1	0.343	42.5	30.4
Beam flange	12	206.85	358.7	564.6	0.475.	38.6	26.9
Stiffener	12	208.35	354.6	571.5	0.458	29.7	28.1
M20 bolt	20	209.58	998.7	1165.7	0.487	9.5	49.8
M24 bolt	24	206.87	976.4	1198.5	0.513	7.6	50.6

Table Note: *t* and *d* are the plate thickness and bolt diameter; *E* is the elastic modulus; *f_y_* and *f_u_* are the yield strength and tensile strength; *ε_y_* and *ε_u_* are the corresponding yield strain and tensile strain; *A* is the elongation after break rate.

**Table 3:** Load history.

***θ_dr_*** **(rad)**	0.00375	0.005	0.0075	0.01	0.015	0.02	0.03	0.04	0.05	0.06	0.07	0.08
**Displacement** **(mm)**	7.785	10.5	15.75	21	31.5	42	63	84	105	126	147	168
***n***	6	6	6	4	2	2	2	2	2	2	2	2

**Table 4:** Typical failure modes of the bolts.

Failure Classification	Failure Mode I	Failure Mode II	Failure Mode III
Destruction surface angle	0°	45°	0–45°
Pattern	Tensile failure	Shear failure	Both failure patterns exist
Phenomenon	Bolt shank breakage and contraction	Bolt shank oblique shear failure	Bolt shank axis bending and necking
Bolt failure location	The first row of specimen EC-EP1 and EC-EP2	The bottom rows of IC-EP2 specimen	The bottom two rows of IC-EP2 specimen
Corresponding figure	Figure 9a,c,d	Figure 9b	Figure 9e

**Table 5:** Main test results.

Specimen		*K_ji_* (kN·m/mrad)	*M_y_* (kN·m)	*M_d_* (kN·m)	*M_p_* (kN·m)	*M_max_* (kN·m)	*θ_max_* (rad)	*θ_y_* (mrad)	*θ_u_* (rad)	*μ_θ_*
IC-EP1	west	55.8	130.4	213.4	195.6	251.1	0.05	6.7	0.06	9.0
	east	57.5	124.1	203.2	186.1	239.1	0.06	6.5	0.06	9.2
IC-EP2	west	63.3	138.6	203.8	208.3	239.8	0.07	7.9	0.07	8.9
	east	62.6	132.1	194.4	198.1	228.7	0.06	7.1	0.06	8.5
EC-EP1	-	25.7	130.7	201.1	195.6	236.6	0.04	4.7	0.05	10.6
EC-EP2	-	27.6	120.1	191.9	180.1	225.8	0.04	5.1	0.05	9.8

Table Note: *K_ji_* is the initial rotational stiffness of the joints; *M_y_, M_d_, M_p_, M_max_, θ_max_, θ_y_*, and *θ_u_* are defined by the key parameters of the connections, *μ_θ_* is the ductility coefficient of the test specimen, *μ_θ_* = *θ_u_*/*θ_y_*.

**Table 6:** Key parameters of energy dissipation.

Specimen		*E_e_*	*ξ_e_*	*W_total_*	Δ (%)
IC-EP1	W	1.73	0.276	191.43	41.6
	E	1.70	0.271	179.60	30.7
IC-EP2	W	2.21	0.352	260.69	59.5
	E	2.22	0.353	252.90	54.7
EC-EP1	-	1.50	0.238	163.44	0
EC-EP2	-	1.35	0.214	139.66	−14.5

Table Note: *E_e_*, *ξ_e_*, and *W_total_* are the energy dissipation coefficient, the equivalent viscous damping coefficient, and the total dissipated energy, respectively, Δ represents the relative difference.

**Table 7:** Bolt and steel material properties.

Material Type	*f_y_*/MPa	*f_u_*/MPa	*ε_y_*/%	E/GPa	E_st_/GPa
Q345B steel	370.16	556.20	0.40	207.27	0.02E
10.9-grade high strength bolt	987.55	1182.10	0.50	208.23	0.11E

**Table 8:** Comparison of the FE analysis and test results.

Specimen	Method	*K_ji_* (kN·m/mrad)	*M_y_* (kN·m)	*θ_y_* (mrad)	*K_FE_/K_Test_*	Method	*K_ji_* (kN·m/mrad)	*M_y_* (kN·m)	*θ_y_* (mrad)	*K_FE_/K_Test_*
IC-EP1/west	Test	55.8	130.4	6.7	-	FE(1/2)	53.5	123.2	5.3	0.96
	FE(all)	57.8	136.7	6.1	1.04	FE(1/4)	52.2	155.8	5.7	0.94
IC-EP2/west	Test	63.3	138.6	7.9	-	FE(1/2)	65.6	150.6	6.7	1.04
	FE(all)	64.9	146.3	7.8	1.03	FE(1/4)	66.1	163.2	5.5	1.04
EC-EP1	Test	25.7	130.7	4.7	-	FE(1/2)	28.5	153.8	4.1	1.11
	FE(all)	26.3	141.3	5.3	1.02					
EC-EP2	Test	27.6	120.1	5.1	-	FE(1/2)	29.8	135.7	3.7	1.08
	FE(all)	30.9	130.5	4.9	1.12					
